# 58993 The Efficacy of Converting an In-person Commercialization Education Course to a Virtual and Flipped Experience

**DOI:** 10.1017/cts.2021.556

**Published:** 2021-03-30

**Authors:** Samantha Cook, Meghan Cuddihy, Sara Risch, Michelle Larkin, Bradley Martin, Jonathan Servoss

**Affiliations:** Michigan Medicine, University of Michigan

## Abstract

ABSTRACT IMPACT: The successful conversion of an in-person biomedical research commercialization education course to a fully virtual and flipped experience (self-paced) allows greater participation from faculty investigators at CTSA institutions and serves as a model for similar educational programs intended to accelerate the translation of biomedical innovations to products of impact. OBJECTIVES/GOALS: Due to COVID-19, University of Michigan’s Fast Forward Medical Innovation developed new educational resources and leveraged virtual learning tools to convert a successful in-person research commercialization course to a fully virtual, flipped format and evaluated the effectiveness of the converted course compared to the in-person equivalent. METHODS/STUDY POPULATION: Two novel interactive modules (intellectual property and FDA regulation) and five instructional videos (customer discovery, value proposition, opportunity sizing, target product profile, and patent searches) were developed while Constant Contact and Zoom were used for a weekly progression of content delivery and to flip the course: (1) forming/testing value propositions, (2) intellectual property, (3) regulatory, (4) medical reimbursement, (5) business case development. A total of 32 faculty and graduate students completed the virtual, flipped course and submitted a post-course evaluation. Results of the converted course were compared to evaluation results from the in-person course. RESULTS/ANTICIPATED RESULTS: Open rates for the weekly email content were: (1)61%, (2)67%, (3)65%, (4)67%, and (5)59 %. Total views for the modules and videos were: IP-28, regulation-19, customer discovery-62, value proposition-21, opportunity sizing-66, target product profile-11, and patent searches-29. Evaluation results from the virtual course (n=22) were compared to mean results from the 5 previous in-person courses (n=42); 86% of virtual course respondents stated the course met the objectives compared to 85% of in-person respondents; 87% of virtual respondents stated the course met their expectations compared to 100% of in-person; 87% of virtual respondents said they would participate in a follow-up program compared to 94% in-person; 91% of virtual respondents would recommend the course to others compared to 97% of in-person. DISCUSSION/SIGNIFICANCE OF FINDINGS: Email open rates and content views suggest positive flipped participation. Overall, the converted course was comparable to the in-person course at meeting objectives, suggesting the virtual format is effective at delivering the course content and holds the potential for engaging a broader audience.

